# Rhabdomyosarcoma Confined to the Bone Marrow: A Case Report and Literature Review

**DOI:** 10.3390/curroncol33060331

**Published:** 2026-06-02

**Authors:** Mohammad Hassan Hodroj, Chloe Batrouni, Alexandre da Silva Faco Junior, Mohammad Amin Salehi, Ramy Saleh

**Affiliations:** 1 Department of Medical Oncology, Cedars Cancer Centre, McGill University Health Centre, Montreal, QC H4A 3J1, Canada; mohammad.hodroj@mail.mcgill.ca (M.H.H.); alexandre.faco@mail.mcgill.ca (A.d.S.F.J.);; 2 Medical Research Volunteer Program (MRVP), American University of Beirut, Beirut 1107-2020, Lebanon; crb10@mail.aub.edu

**Keywords:** rhabdomyosarcoma, bone marrow, PET scan, thrombocytopenia, anemia

## Abstract

Rhabdomyosarcoma (RMS) confined to the bone marrow represents an exceptionally rare and aggressive presentation that can mimic primary hematological malignancies, often leading to diagnostic delays and therapeutic challenges. We report the case of a 34-year-old woman who presented with clinical and laboratory findings highly suggestive of a hematological disorder, including cytopenias and diffuse bone marrow involvement. Initial evaluation raised suspicion for leukemia; however, comprehensive diagnostic work-up, including immunohistochemistry and molecular studies, ultimately confirmed the diagnosis of *PAX3/FOXO1* gene-rearranged alveolar RMS isolated in the bone marrow, with no identifiable primary soft tissue mass. The patient was treated with an intensive multi-agent chemotherapy regimen, resulting in a marked hematological recovery and a significant radiological improvement after a limited number of cycles. We further reviewed the limited literature on bone-marrow-confined RMS, highlighting the proposed pathophysiological mechanisms, diagnostic pitfalls, and reported treatment strategies. Given the absence of standardized management guidelines for this rare entity, therapeutic approaches are often extrapolated from conventional RMS protocols or regimens used for high-grade sarcomas. Our experience supports the potential efficacy of intensive chemotherapy in achieving meaningful clinical responses. This case report emphasizes the challenges in the diagnosis of RMS confined to the bone marrow due to its atypical presentation. It also highlights the poor prognosis and aggressiveness of this entity compared to conventional RMS.

## Simple Summary

Rhabdomyosarcoma (RMS) confined to the bone marrow is an extremely rare, aggressive RMS subtype that can mimic hematologic malignancies, making its diagnosis challenging. In this article, we report the case of a 34-year-old woman who presented with features suggestive of hematological malignancies and was ultimately diagnosed with *PAX3/FOXO1* gene-rearranged alveolar RMS confined to the bone marrow. The patient received intensive chemotherapy with a significant hematological and radiological response to treatment after a few cycles. This case report highlights the challenge of diagnosing this subtype of RMS, the available data in the literature, and the comparison of available chemotherapy regimens with our treatment approach.

## 1. Introduction

Rhabdomyosarcoma (RMS) is a rare and highly aggressive malignant neoplasm arising from primitive mesenchymal cells committed to skeletal muscle differentiation. Although it most commonly originates in skeletal muscle, RMS may also develop in a variety of soft tissues, including connective, adipose, and fibrous tissues, reflecting the pluripotent nature of its cell of origin. Histologically, RMS comprises several distinct subtypes, most notably embryonal, alveolar, pleomorphic, and spindle-cell/sclerosing variants, each characterized by unique molecular features, clinical behavior, and prognostic implications [1]. Several subtypes of RMS have been described in the literature (Table 1). Among these subtypes, the embryonal subtype is the most frequently encountered, particularly in pediatric populations, and is generally associated with a more favorable prognosis. In contrast, alveolar RMS, often defined by recurrent translocations such as *PAX3–FOXO1* or *PAX7–FOXO1*, and pleomorphic RMS, seen predominantly in adults, tend to exhibit a more aggressive clinical course and inferior survival outcomes [2].

RMS is primarily a disease occurring in childhood and adolescence, accounting for approximately 4.5% of all pediatric malignancies and representing the most common soft tissue sarcoma in children [8]. Its incidence sharply declines with age, and, in adults, RMS is exceedingly rare, constituting less than 1% of all malignancies [9]. Notably, adult RMS often demonstrates distinct clinical and biological characteristics compared with its pediatric counterpart, including a higher prevalence of unfavorable histological subtypes and poorer overall outcomes. RMS can arise in virtually any anatomic location; however, common primary sites include the head and neck region, genitourinary tract, retroperitoneum, and extremities. Its clinical presentation varies widely depending on tumor location, size, and extent of disease, ranging from localized mass effects to symptoms related to metastatic spread [10].

While RMS typically presents as a primary soft tissue mass with or without metastatic involvement, rare and atypical presentations have been described. One of the most unusual forms is RMS confined exclusively to the bone marrow, without an identifiable primary tumor elsewhere in the body. This entity was first reported in 1988 by De La Serna et al., who described patients in which malignant rhabdomyoblastic cells were detected solely within the bone marrow compartment [11]. Since then, only a limited number of similar patients have been documented, underscoring the rarity and diagnostic complexity of this presentation. The pathogenesis of marrow-confined RMS remains poorly understood, and it is unclear whether these cases represent occult primary tumors with early dissemination or a distinct biological entity [12].

Clinically, RMS involving the bone marrow often mimics hematological malignancies such as acute leukemia or lymphoma. Patients may present with nonspecific systemic symptoms, including fatigue, fever, weight loss, and bone pain, alongside laboratory abnormalities such as anemia, thrombocytopenia, and leukopenia resulting from the marrow infiltration and suppression of normal hematopoiesis [12]. Peripheral blood findings may further confound the diagnosis, occasionally demonstrating circulating immature cells. In such scenarios, bone marrow aspiration and biopsy become essential diagnostic tools. Histopathological evaluation, supported by immunohistochemistry (e.g., desmin, myogenin, and MyoD1) and molecular studies, is critical to distinguish RMS from other small round blue cell tumors and hematological neoplasms [2].

Given its rarity, aggressive behavior, and diagnostic challenges, RMS confined to the bone marrow represents a clinically significant entity that requires a high index of suspicion for accurate and timely diagnosis [12]. In this article, we report the case of a 34-year-old woman who presented with clinical and laboratory features suggestive of a hematological malignancy and was ultimately diagnosed with RMS isolated in the bone marrow, highlighting the importance of considering this rare diagnosis in the differential diagnosis of marrow-infiltrative disorders.

## 2. Case Presentation

**Figure 1:**
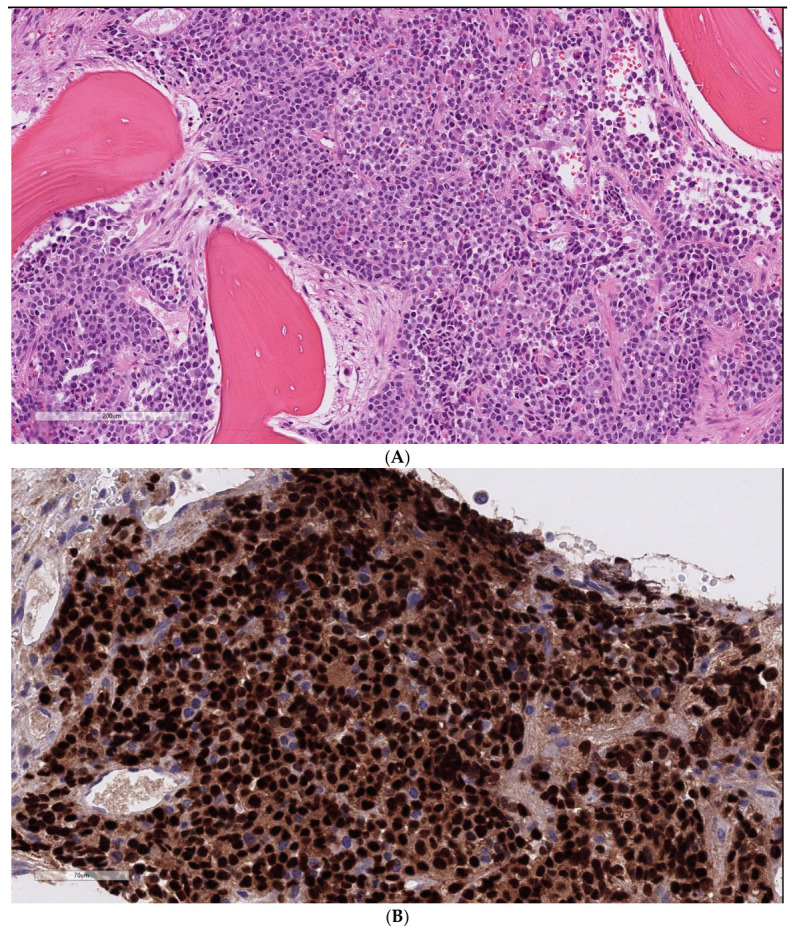
(**A**) Histopathological examination with H & E staining. (**B**) Histopathological examination with myogenin staining showing >99% positivity.

**Figure 2:**
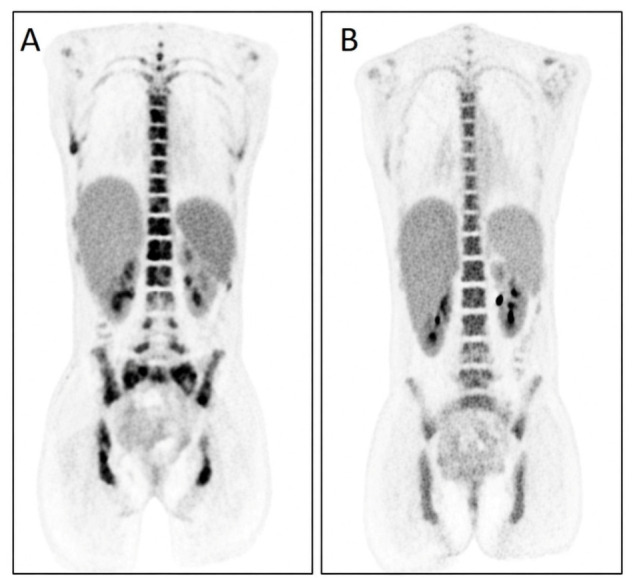
(**A**) Baseline PET scan showing a diffuse intense radiotracer uptake in the bone marrow of the appendicular and axial skeletons. (**B**) PET scan performed in December 2025 showing a remarkable reduction in radiotracer avidity post-treatment.

A 34-year-old woman, without previous medical history, presented to the emergency room in September 2025 with severe fatigue of a few weeks’ duration and recent episodes of easy bruising. Physical examination demonstrated marked pallor and widespread bruises, predominantly on the extremities. The patient was conscious, cooperative, and presented with tachycardia and borderline blood pressure. Blood tests revealed severe anemia with a hemoglobin level of 69 g/L, a thrombocytopenia of 6 × 10^9^ g/L, disseminated intravascular coagulopathy (DIC) with a fibrinogen level of 0.38 g/L, and an International Normalized Ratio (INR) of 1.38. Otherwise, all other laboratory parameters, including electrolytes and liver and kidney function, were within normal ranges. The patient developed oozing and bleeding around sites of intravenous lines. She received intravenous vitamin K and transfusions of packed red blood cells, platelets, fresh frozen plasma, and cryoprecipitate. A hematological malignancy was suspected, and a bone marrow biopsy was performed. Pathological examination showed RMS with a *PAX3/FOXO1* gene fusion, without evidence of lymphoma or leukemia. Histopathological examination using H & E staining is depicted in Figure 1A and that with myogenin staining (>99% positive), which is typical and highly specific for RMS, is depicted in Figure 1B. An FDG-PET scan was performed to locate the primary tumor and assess the extent of the disease. However, this scan showed diffuse intense uptake in the bone marrow of the appendicular and axial skeleton, as well as splenomegaly without evidence of radiotracer avidity. There was no evidence for any primary tumor on the PET scan (Figure 2A). Based on clinical presentation, laboratory findings, and imaging, the patient was diagnosed with *PAX3/FOXO1*-fused RMS confined to the bone marrow and was admitted for treatment. Treatment was initiated with dactinomycin, vincristine, and cyclophosphamide (VDC) on days 1 and 8, with a significant improvement in blood test results by the end of the first cycle. Hemoglobin increased to 85 g/L, platelet count increased to 57 × 10^9^ g/L, and fibrinogen and the INR normalized without the need for additional transfusions. The patient reported a remarkable improvement in fatigue and energy levels post the first cycle, which were maintained after the second cycle, with hemoglobin stabilizing above 80 g/L and platelets spontaneously normalizing to 190–200 × 10^9^/L. Upon the improvement in her blood tests, the treatment was augmented to include ifosfamide and etoposide (IE) alternating with VDC, which is an indicated treatment option for some advanced sarcomas [13]. She completed a total of three cycles of VDC and two cycles of IE with a repeat PET scan in December 2025, showing a remarkable reduction in radiotracer uptake throughout the axial and proximal appendicular skeleton with only mild uptake corresponding to the stable diffuse sclerosis of the marrow space (Figure 2B). Her hemoglobin started to increase, reaching 105 g/L in December 2025 and then 121 g/L in March 2026, with a normal complete blood count. The patient received an additional third cycle of IE in January 2026, and following the significant improvement in her blood counts, she was maintained on VDC only.

## 3. Discussion

RMS predominantly affects the pediatric population, with the highest incidence occurring in children under 10 years of age. However, RMS confined to the bone marrow is a rare disease, with only five pediatric patients (ages 15–18 years) and four adult patients (ages 52–64 years) reported in the literature [11,12,14,15,16,17,18,19]. Our patient is a 34-year-old adult woman who does not fall within the reported age group of adult patients. Similar to seven of the reported patients, our patient had the alveolar subtype of RMS, which commonly harbors the *PAX3–FOXO1* fusion [12,14,15,16,17,18]. In addition, our patient’s presentation was typical of hematological malignancies, as reported in most of the described case reports, including fatigue, anemia, thrombocytopenia, and even DIC, as in patient 8 (Table 2). On the contrary, patients 2 and 3 presented with nonspecific symptoms, and patient 9 presented with hypercalcemia. Treatment approaches among the reported patients with RMS confined to the bone marrow were heterogeneous, reflecting both the rarity of the condition and the absence of standardized management strategies. Most patients received multi-agent chemotherapy regimens commonly used for high-risk or metastatic RMS, frequently including vincristine, cyclophosphamide, dactinomycin, ifosfamide, etoposide, or doxorubicin. Similar to the treatment reported in patient 4, VDC was initiated, with rapid symptom improvements observed within 2 weeks. Given the known aggressiveness of this subtype, treatment was augmented by alternating IE with VDC after blood count stabilization. The administered regimen resulted in a remarkable improvement in blood counts and imaging results. Alternating VDC/IE has been described in several pilot studies as an effective treatment for distinct advanced sarcoma subgroups, including RMS, undifferentiated sarcomas, Ewing sarcoma, and other small round cell sarcomas [13,20]. Other patients described different treatment regimens, including those for acute lymphoblastic leukemia (as for patient 8), bone marrow transplantation, steroids, and VDC with doxorubicin [12,14,19]. Among the reported patients of RMS confined to the bone marrow, clinical outcomes have been variable, ranging from death due to disease to partial response to therapy. Of the five previously reported pediatric patients, four died from progressive disease within 3 to 10 months of diagnosis, while the outcome was not specified in one patient. Only one reported pediatric patient achieved a partial response. Specifically among adult patients, two of the four reported patients achieved a partial response, whereas the remaining two patients died due to the disease within 2 to 12 months. Similar to the previously reported adult patients with favorable outcomes, our patient has achieved a partial response and remains alive with disease showing ongoing response at 8 months of follow-up to date. It was observed that the VDC regimen was the common regimen in three of the four patients that achieved a partial response, including our patient (Table 2).

The accurate underlying mechanisms of RMS confined to the bone marrow remain unclear. A series of hypotheses have been proposed to contribute to the development of this subtype. The specific tropism of the bone marrow has been discussed, in which tumor cells express adhesion molecules and chemokine receptors (e.g., CXCR4), particularly the alveolar rhabdomyosarcoma subtype [21]. Similarly, CXCR4, specifically CXCL12-CXCR4, has been highly associated with regulating cancer progression in the bone marrow by creating a tumor microenvironment that results in cancer metastasis [22]. Additionally, Ewing sarcoma, which is an aggressive round cell sarcoma mainly affecting the bones, is known to be rich in CXCR4, leading to an increased migration and invasion capacity [23]. Another proposed mechanism is the transformation of primitive mesenchymal progenitor cells already present in the bone marrow into RMS cells via a common lineage of origin. Moreover, *PAX3-FOXO1* or *PAX7-FOXO1* fusions in alveolar RMS are considered aggressive molecular drivers that promote dissemination to the bone marrow [12].

Another significant aspect that should be highlighted in RMS confined to the bone marrow is the use of a suitable imaging modality for diagnosis. Several articles have discussed different imaging modalities. However, the PET/CT scan remains a valuable, sensitive, non-invasive tool to map the entire skeleton, detect occult metastases, and guide treatment decisions in RMS, especially in the alveolar subtype that frequently metastasizes to the bone marrow [16]. The PET/CT scan with FDG has been shown to be superior to the bone scan for detecting bone metastasis at staging. In addition, in the subtype confined to the bone marrow, the PET/CT scan not only confirms the absence of a primary tumor and the infiltration of the bone marrow but also is used as a sensitive follow-up tool to compare the radiotracer uptake in the bone marrow post-treatment [24].

## 4. Conclusions

RMS confined to the bone marrow without evidence of primary tumors remains a rare entity that presents in an advanced stage and appears to be mostly an alveolar subtype with a *PAX3/FOXO1* gene fusion. Given its ability to mimic hematological malignancies, this aggressive subtype should be rapidly diagnosed, considered a metastatic disease, and directly treated with systemic therapy. The nine reported patients in the literature and our patient with RMS confined to the bone marrow, demonstrated significant diagnostic challenges due to the atypical presentation of the disease. Overall, these patients exhibited an aggressive clinical course and poor prognosis, including those who initially responded to therapy, compared with patients with conventional RMS presenting with an identifiable primary tumor site. 

## Figures and Tables

**Table 1:** A summary of the major rhabdomyosarcoma (RMS) subtypes, including their characteristic histopathological features, predominant genetic alterations, molecular signatures, typical clinical presentation, and prognostic implications.

Subtypes	Key Genetic Features	Characteristics
**Alveolar RMS (ARMS)** [3]	*PAX3–FOXO1* or *PAX7–FOXO1* fusion (t(2;13), t(1;13))	Strongly associated with metastasis and poor prognosis
**Embryonal RMS (ERMS)** [4]	Loss of heterozygosity at 11p15; RAS pathway mutations (*NRAS, KRAS, HRAS*); TP53 alterations (subset)	Most common subtype; better prognosis than ARMS
**Spindle-cell/sclerosing RMS** [5,6]	*MYOD1* mutations (L122R); *VGLL2* and *NCOA2* fusions (especially in infants)	Heterogeneous; MYOD1-mutant characterized by aggressive behavior
**Pleomorphic RMS** [7]	Complex karyotype; multiple chromosomal gains/losses; TP53 mutations common	More prevalent in adults; resembles high-grade undifferentiated sarcoma
**Fusion-negative RMS (FN-RMS)** [4]	Overlaps with ERMS; RAS pathway mutations; FGFR4 and PIK3CA alterations	Biologically distinct from fusion-positive RMS; better prognosis

**Table 2:** A summary of reported patients with rhabdomyosarcoma confined to the bone marrow in the literature.

Patient #	Author et al. (Year)	Gender	Age (years)	Subtype of Rhabdomyosarcoma	Presenting Symptoms	Treatment/Management	Outcome (Months After Diagnosis)
**1**	Present Case (2026)	Woman	34	Alveolar (PAX3/FOXO1)	Fatigue, anemia, thrombocytopenia, DIC.	Chemotherapy started with dactinomycin, vincristine, and cyclophosphamide, and then escalated to vincristine, ifosfamide, and etoposide.	Alive with disease, PR (8)
**2**	Affinita et al. (2022) [12]	Man	15.7	Alveolar (PAX3/FOXO1)	Nonspecific symptoms.	Chemotherapy and allogeneic bone marrow transplantation (ABMT).	DOD (10)
**3**	Affinita et al. (2022) [12]	Man	16.7	Alveolar (PAX3/FOXO1)	Nonspecific symptoms.	Chemotherapy and ABMT (post-PD).	Death due to toxicity (6)
**4**	Kaur et al. (2020) [14]	Woman	64	Alveolar	Generalized weakness, ecchymotic patches, lower backache, and anemia for 20 days and fever for three days; similar symptoms to those of acute leukemia.	Chemotherapy with vincristine, dactinomycin, and cyclophosphamide; the patient was transfusion-independent for 15 days.	Alive with disease (not specified)
**5**	López-Andrade et al. (2019) [15]	Woman	56	Alveolar	Symptoms mimicked those of acute lymphoblastic leukemia; persistent back pain and epistaxis.	No treatment as the patient returned to their home country for treatment and passed away 2 months after diagnosis.	DOD (2)
**6**	Karagiannis et al. (2015) [16]	Woman	61	Alveolar	Shortness of breath, anorexia, and fatigue; symptoms mimicked a hematological disease with pancytopenia.	At first, topotecan/cyclophosphamide and blood transfusions, then vinorelbine.	Alive with disease, PR (7)
**7**	Kern et al. (2015) [17]	Woman	52	Alveolar	Fatigue, thrombocytopenia, easy bruising, and a petechial rash; mimicked acute leukemia.	Steroids for presumed idiopathic thrombocytopenia; treatment with chemotherapy.	DOD (12)
**8**	Sandberg et al. (2001) [18]	Man	12	Alveolar	Systemic symptoms similar to acute leukemia, anemia, thrombocytopenia, and DIC manifestations; history of abdominal pain, vomiting, and fever for one and a half weeks.	Chemotherapy consisted of methotrexate, prednisone, vincristine, and asparaginase, as well as supportive therapy.	DOD (3)
**9**	Morandi et al. (1996) [19]	Woman	18	Not specified	Symptoms similar to those of acute leukemia; a few days’ history of easy bruising, pallor, and fatigue.	Monthly courses of alternating VAC-VAdrC (V, vincristine; A, dactinomycin; C, cyclophosphamide; Adr, doxorubicin).	DOD (not specified)
**10**	De La Serna et al. (1988) [11]	Man	18	Not specified	Anemia, renal failure, lower back pain, sternum tenderness, thrombocytopenia, leucoerythroblastic blood film, and hypercalcemia.	Furosemide and calcitonin were used to treat hypercalcemia and renal failure; intensive chemotherapy, including vincristine, actinomycin D, cyclophosphamide, and doxorubicin; only in the beginning, non-steroidal anti-inflammatory drugs were used (but they made the patient worse).	Alive with disease, PR (5)

PR: partial response; DOD: died of disease; DIC: disseminated intravascular coagulation.

## Data Availability

Data are available from the corresponding author upon request.
